# Above-Curie-temperature ultrafast terahertz emission and spin current generation in a 2D superlattice (Fe_3_GeTe_2_/CrSb)_3_

**DOI:** 10.1093/nsr/nwae447

**Published:** 2024-12-11

**Authors:** Peiyan Li, Na Wu, Shanshan Liu, Yu Cheng, Piming Gong, Junwei Tong, Jianan Liu, Wei He, Faxian Xiu, Jimin Zhao, Sheng Meng, Xiaojun Wu

**Affiliations:** Hangzhou International Innovation Institute, Beihang University, Hangzhou 311115, China; School of Electronic and Information Engineering, Beihang University, Beijing 100191, China; Beijing National Laboratory for Condensed Matter Physics, Institute of Physics, Chinese Academy of Sciences, Beijing 100190, China; School of Physical Sciences, University of Chinese Academy of Sciences, Beijing 100049, China; State Key Laboratory of Surface Physics and Department of Physics, Fudan University, Shanghai 200433, China; Beijing National Laboratory for Condensed Matter Physics, Institute of Physics, Chinese Academy of Sciences, Beijing 100190, China; School of Physical Sciences, University of Chinese Academy of Sciences, Beijing 100049, China; Beijing National Laboratory for Condensed Matter Physics, Institute of Physics, Chinese Academy of Sciences, Beijing 100190, China; School of Physical Sciences, University of Chinese Academy of Sciences, Beijing 100049, China; Department of Physics, Freie Universität Berlin, Berlin 14195, Germany; Beijing National Laboratory for Condensed Matter Physics, Institute of Physics, Chinese Academy of Sciences, Beijing 100190, China; School of Physical Sciences, University of Chinese Academy of Sciences, Beijing 100049, China; Beijing National Laboratory for Condensed Matter Physics, Institute of Physics, Chinese Academy of Sciences, Beijing 100190, China; School of Physical Sciences, University of Chinese Academy of Sciences, Beijing 100049, China; State Key Laboratory of Surface Physics and Department of Physics, Fudan University, Shanghai 200433, China; Beijing National Laboratory for Condensed Matter Physics, Institute of Physics, Chinese Academy of Sciences, Beijing 100190, China; School of Physical Sciences, University of Chinese Academy of Sciences, Beijing 100049, China; Songshan Lake Materials Laboratory, Dongguan 523808, China; Beijing National Laboratory for Condensed Matter Physics, Institute of Physics, Chinese Academy of Sciences, Beijing 100190, China; School of Physical Sciences, University of Chinese Academy of Sciences, Beijing 100049, China; Songshan Lake Materials Laboratory, Dongguan 523808, China; Hangzhou International Innovation Institute, Beihang University, Hangzhou 311115, China; School of Electronic and Information Engineering, Beihang University, Beijing 100191, China; Zhangjiang Laboratory, Shanghai 201210, China; Wuhan National Laboratory for Optoelectronics, Huazhong University of Science and Technology, Wuhan 430074, China

**Keywords:** ultrafast terahertz spin current, 2D superlattice (Fe_3_GeTe_2_/CrSb)_3_, laser-enhanced proximity effect, above Curie temperature

## Abstract

The increasing demand for denser information storage and faster data processing has fueled a keen interest in exploring spin currents up to terahertz (THz) frequencies. Emergent 2D intrinsic magnetic materials constitute a novel and highly controllable platform to access such femtosecond spin dynamics at atomic layer thickness. However, the function of 2D van der Waals magnets are limited by their Curie temperatures, which are usually low. Here, in a 2D superlattice (Fe_3_GeTe_2_/CrSb)_3_, we demonstrate ultrafast laser-induced spin current generation and THz radiation at room temperature, overcoming the challenge of the Curie temperature of Fe_3_GeTe_2_ being only 206 K. In tandem with time-resolved magneto-optical Kerr effect measurements and first-principles calculations, we further elucidate the origin of the spin currents—a laser-enhanced proximity effect manifested as a laser-induced reduction of interlayer distance and enhanced electron exchange interactions, which causes transient spin polarization in the heterostructure. Our findings present an innovative, magnetic-element-free route for generating ultrafast spin currents within the 2D limit, underscoring the significant potential of laser THz emission spectroscopy in investigating laser-induced extraordinary spin dynamics.

## Teaser

First demonstration of above-Curie-temperature ultrafast THz spin current generation in 2D magnetic materials, with first-principles calculations revealing a laser-enhanced proximity effect as the origin of the spin currents.

## INTRODUCTION

Ultrafast spin currents have developed into promising information carriers, revolutionizing the landscape of high-speed and energy-efficient spintronic devices [1–6]. This burgeoning research field traces its origins back to the seminal observation of subpicosecond demagnetization in a nickel film triggered by laser pulses [1] and has since been propelled by a series of fundamentally intriguing ultrafast magnetic processes [7–14]. To delve into these magnetization dynamics, several effective probe techniques have been employed, including the time-resolved magneto-optical Kerr effect (TRMOKE) [15,16], X-ray magnetic circular dichroism [14] and photoemission spectroscopy [12,17]. In addition to these methods, terahertz (THz) emission spectroscopy is another well-established scheme that provides insights into transient spin dynamics with subpicosecond time resolution [18–20]. It is widely utilized in 3D magnets as an ultrafast, sensitive and contactless amperemeter for spin currents [18–25]. By contrast, the recent emergence of 2D intrinsic magnetic materials with remarkable properties [26–35]—such as highly tunable characteristics and sensitive interlayer coupling—creates exciting new possibilities for exploring non-equilibrium spin dynamics and coherent THz pulses down to the atomically thin limit [21]. This development has the potential to bring about key advancements in low-dimensional THz spintronic devices for future storage and quantum information applications.

However, realizing ultrafast spin currents in 2D magnetic materials at room temperature is extremely challenging and thus rarely reported. The long-range ferromagnetic order of van der Waals crystals is vulnerable to thermal fluctuations and is only established at low temperatures [27–30]. Despite recent studies revealing room-temperature spintronic THz emission in a 2D ferromagnetic/topological insulator heterostructure [21] due to interface-enhanced Curie temperature (*T*_C_) up to 400 K [36], the generation of THz spin currents above a finite *T*_C_ is a more critical issue that remains elusive.

Here, we first demonstrate the generation of above-*T*_C_ ultrafast THz spin currents based on 2D magnetic materials. Specifically, we investigate a 2D layered metallic ferromagnetic/antiferromagnetic superlattice (Fe_3_GeTe_2_/CrSb)_3_ (abbreviated as (FGT/CS)_3_) with an intrinsic *T*_C_ of 206 K [35]. Upon photoexcitation by ultrafast optical pulses, we successfully observed room-temperature ultrafast spin current generation and coherent THz emission in the (FGT/CS)_3_ superlattice. Combined with the first-principles calculation based on the real-time density functional theory framework (rt-TDDFT) and Ehrenfest Molecular Dynamics, we attribute the underlying physics mechanism of the spin current generation to a laser-enhanced magnetic proximity effect at the interface. Our understanding is corroborated by the TRMOKE measurement of the corresponding transient spin polarization.

## RESULTS

### Above-*T*_C_ spintronic THz emission from the (FGT/CS)_3_ superlattice

**Figure 1.**
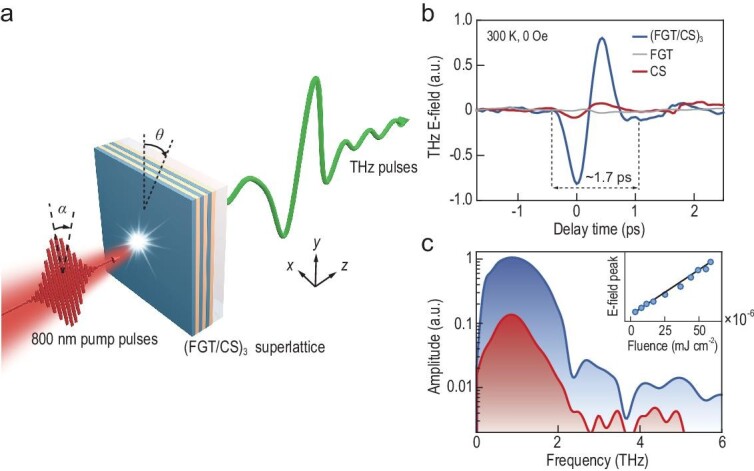
THz emission from the (FGT/CS)_3_ superlattice at room temperature without external magnetic fields. (a) Schematic illustration of the THz emission from the (FGT/CS)_3_ superlattice. (b) THz emission temporal waveforms of the (FGT/CS)_3_ superlattice (blue curve), FGT (gray curve) and CS (red curve). (c) Corresponding Fourier-transformed spectra of (b). Inset: pump fluence dependence of radiated THz electric-field strength from the superlattice. a.u.: arbitrary units.

The (FGT/CS)_3_ superlattice is constructed by repeating the FGT/CS heterostructure for three periods on mica substrate using molecular beam epitaxy (MBE). Each FGT/CS heterostructure consists of four layers of Fe_3_GeTe_2_ (FGT, 3.2 nm) and a single layer of CrSb (CS, 1.6 nm) (for more details see Section S1). A schematic overview of the THz emission measurement is delineated in Fig. 1a. Vertically polarized (along the *y*-axis) 800-nm femtosecond laser pulses with a pump fluence of 3.75 × 10^−5^ mJ/cm^2^ are used to excite the (FGT/CS)_3_ superlattice at room temperature. Meanwhile, a photoconductive antenna is configured to detect THz signals copropagating with the laser beam. Figure 1b illustrates a typical THz temporal waveform of the (FGT/CS)_3_ superlattice, with a duration of ∼1.7 ps. Under similar experimental settings, however, the detected THz radiation from the CS-only film (4 nm) is nearly an order of magnitude weaker, and the THz radiation from the FGT film (10 nm) is barely detectable. The corresponding Fourier transformation results are shown in Fig. 1c. Additionally, the THz electric-field peak intensity is observed to be linearly proportional to the pump fluence, as depicted in the inset of Fig. 1c. These findings indicate that the predominant THz emission from the (FGT/CS)_3_ superlattice does not originate solely from CS-only or FGT-only films.

**Figure 2.**
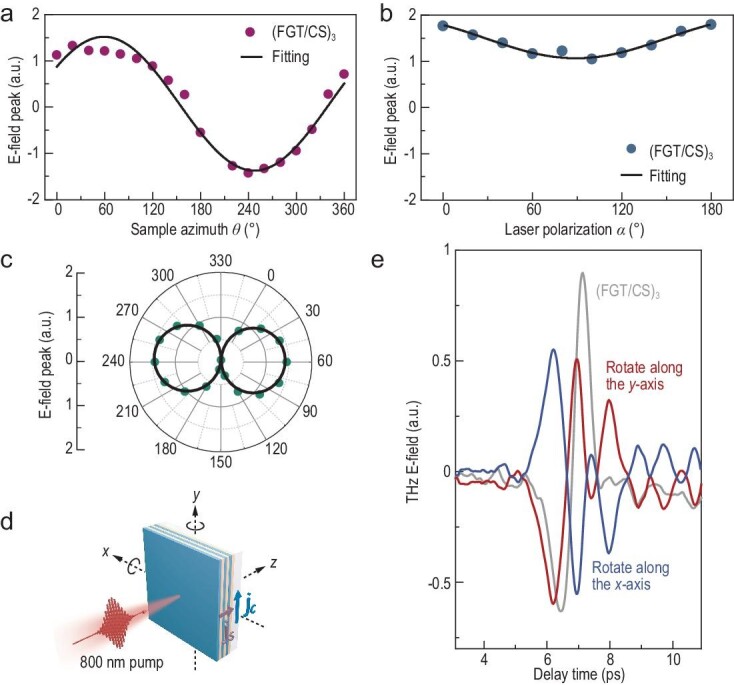
Verification of the spin-to-charge conversion effect as the predominant THz radiation mechanism from the (FGT/CS)_3_ superlattice_._ (a) The relationship between the THz electric-field peak and sample azimuth $\theta $ is a sinusoidal variation characterized by a period of 360°. (b) The peak-to-peak value of waveforms as a function of laser polarization $\alpha $ for the superlattice. Only a small fraction of THz radiation is related to $\alpha $. (c) The relationship between the laser-polarization-independent component of the superlattice and $\theta $. (d) Temporal THz waveforms from the superlattice were measured by rotating 180° along the *x*- and *y*-axis, which indicate (e) the directions of both spin currents $\overrightarrow {{{j}_s}} $ and charge currents $\overrightarrow {{{j}_c}} $.

To investigate the radiation mechanism, we examined the dependence of THz emission from the (FGT/CS)_3_ superlattice on the sample azimuth angle $\theta $ and the linear laser polarization angle $\alpha $ [21,37]. In Fig. 2a, the relationship between the peak value of the THz waveforms and $\theta $ is plotted, with the laser polarization fixed along the *y*-axis (${\mathrm{\alpha = \ }}{{{\mathrm{0}}}^{\mathrm{\circ }}}$). The THz electric-field peak reaches its maximum value at ${\mathrm{\theta \ = \ 6}}{{{\mathrm{0}}}^{\mathrm{\circ }}}$ and its minimum value at ${\mathrm{\theta \ = \ 24}}{{{\mathrm{0}}}^{\mathrm{\circ }}}$, fitting well by a sinusoidal function with a period of 360°. Subsequently, with $\theta $ fixed at 60°, the dependence of the THz amplitude on the linear laser polarization angle $\alpha $ is summarized in Fig. 2b. As $\alpha $ is varied from 0° to 180°, the THz electric-field peak as a function of $\alpha $ demonstrates a cosine oscillation with a small amplitude, alongside a significant non-zero offset. This outcome shows that only a minor fraction of the THz radiations are associated with laser polarization, while most remain independent (for the influence of laser polarization state on THz emission see Section S3). The corresponding fitted curve, based on a cosine function with a period of 180°, aligns well with the experimental data. Notably, the fitting results in Fig. 2a and b allow us to infer the variation of the laser-polarization-independent THz radiation component as $\theta $ increases from 0° to 360° (Fig. 2c). This component exhibits 2-fold rotational symmetry and is only slightly less than the total THz radiation. Simultaneously, the small-amplitude polarization-dependent contribution, as illustrated in Fig. S2e, corresponds to the total radiation of CS-only films.

Based on the above characterizations, it is feasible to deduce that the dominant component of the generated THz radiation is azimuth-dependent but laser-polarization-independent, thereby ruling out the contribution of the inverse Faraday effect in metallic materials [38] and non-linear optical processes in the crystal [39]. To further elucidate the main THz emission mechanism, we examine the THz radiation under different experimental geometries. As depicted in Fig. 2d and e, the THz polarity reverses when the sample is rotated by 180° along the *x*-axis, with a variation in amplitude due to the dissimilar propagation of 800-nm laser pulses and THz waves through the substrate. In contrast, upon left-right flipping along the *y*-axis, the polarity of the THz waveform remains unchanged. The contribution from magnetic dipole oscillations is therefore negligible [40–42], and these outcomes are entirely consistent with the signatures of the THz radiation induced by the spin-to-charge conversion effect [37], which can be described by:


(1) \begin{eqnarray*}
{\overrightarrow{E_{\rm THz}} \propto \overrightarrow{j_{\rm c}} \propto {\rm\gamma }\cdot{\overrightarrow{j_{\rm s}}}\times } \frac{\overrightarrow{M}}{|M_{\rm s}|},
\end{eqnarray*}

where $\overrightarrow {{{j}_s}} $ represents the ultrafast spin currents driven by femtosecond laser pulses. Utilizing the spin-to-charge conversion effect, the spin currents $\overrightarrow {{{j}_s}} $ can be quantitatively analyzed by recording the electric-field waveform of emitted THz radiation following the decaying charge current $\overrightarrow {{{j}_c}} $. ${\mathrm{\gamma }}$ is the spin Hall angle. $\vec{M}$ denotes the in-plane magnetization, which may be attributed to the interfacial exchange bias [37] rather than external magnetic fields, and ${{{\mathrm{M}}}_{\mathrm{s}}}$ is the saturation magnetization. Therefore, the polarization direction of linearly polarized THz waves can be controlled by rotating the sample along its azimuthal axis. Moreover, when the (FGT/CS)_3_ superlattice is flipped, the direction of the spin current reverses. However, the in-plane magnetization direction remains unchanged or reversed after flipping, which consequently results in the opposite polarity of the emitted THz electric fields.

**Figure 3.**
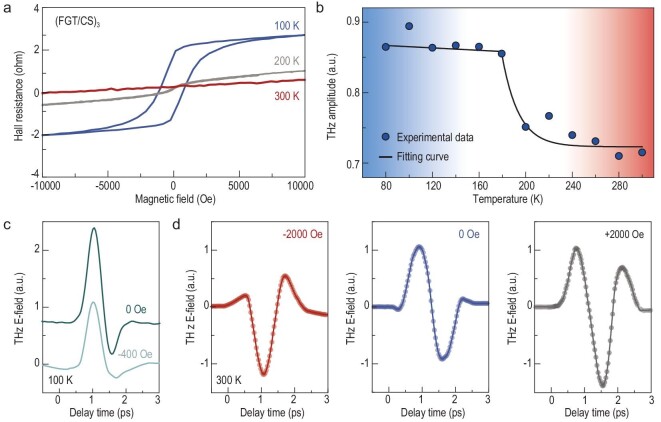
Temperature- and magnetic-field-dependent spintronic THz emission. (a) The anomalous Hall effect results of (FGT/CS)_3_ superlattice under perpendicular geometry at 100 K, 200 K and 300 K. (b) Radiated THz amplitudes as a function of the temperature. (c) At 100 K, THz waveforms emitted from the superlattice were measured under zero magnetic field and with an out-of-plane magnetic field of 400 Oe. (d) At 300 K, applying an out-of-plane magnetic field of +2000 Oe reverses the THz signal. When subsequently flipping the magnetic field, the THz radiation polarity remains constant, but the intensity surpasses that observed without magnets.

Although the above analysis of the THz radiation is bolstered by several consistencies confirming that the predominant THz radiation is attributed to spin-to-charge conversion, prior research on the (FGT/CS)_3_ superlattice [35] introduces two paradoxes challenging our view of spintronic THz emission:

- Where do the spin currents originate from? The *T*_C_ of our superlattice sample is 206 K [35], which is significantly below room temperature. Anomalous Hall effect measurements in Fig. 3a reveal distinguishable hysteresis up to 200 K, which vanishes at 300 K. The laser-induced dissipation of spin angular momentum associated with ultrafast demagnetization [1] is unlikely to occur at room temperature. We cryogenically cooled the sample and measured the temperature dependence of THz radiation. As depicted in Fig. 3b, the THz intensity remains constant at lower temperatures but decreases at 200 K. When the temperature rises above 200 K, the signal intensity stabilizes again. The THz waveforms and corresponding fast-Fourier-transformed spectra are displayed in Fig. S5b and c, where we propose that there are different mechanisms for spin current generation at temperatures below 200 K and above 200 K (for more information see Sections S5–6). Below 200 K, the superlattice exhibits a ferromagnetic phase. The sudden heating of the sample by the femtosecond pumping pulse leads to ultrafast demagnetization, generating spin currents. Above 200 K, ultrafast spin currents can be generated via an unidentified mechanism related to photoexcitation, which will be investigated in subsequent sections using TRMOKE techniques and theoretical analysis.
- The other paradox concerns the spin orientation. Below *T*_C_, the superlattice exhibits anisotropic magnetic properties, with its easy axis along the out-of-plane direction and its hard axis situated within the in-plane direction. This illustrates the fact that the spin orientation should predominantly align along the out-of-plane direction, which theoretically forbids the THz waves propagating along the *z*-axis [18]. He *et al.* have reported that the spin orientation of Cr:(Bi, Sb)_2_Te_3_/CrSb heterostructures is not completely perpendicular to the sample plane without external out-of-plane magnets [43]. This indicates that our superlattice may also exhibit tilted spin orientation, which contains in-plane spin components. To verify this hypothesis, we initially applied a 400 Oe magnetic field parallel to the out-of-plane magnetization (along the surface normal) at 100 K. The intensity of THz radiation is reduced by 42%, as shown in Fig. 3c. Intriguingly, when a perpendicular 2000 Oe external magnetic field is applied at 300 K, the polarity of the THz waveform is reversed. With the subsequent switch of the magnetic field, the THz radiation polarity recovers, but the intensity is enhanced compared to its initial intensity (Fig. 3d). The two observations mentioned above, namely the dependence of THz amplitude on the applied magnetic fields at 100 K and 300 K, respectively, confirm the tilting of spin orientation. Note that the out-of-plane magnetic field strength used for our experiments conducted at both room and low temperatures was consistently applied at 2000 Oe. However, at 100 K, due to the hindrance posed by the cryostat cavity, the magnetic field strength applied to the superlattice was reduced to 400 Oe. Furthermore, as depicted in Fig. S9, the THz amplitude exhibits minimal change under in-plane magnetic fields (2000 Oe), corresponding to the hard axis of the superlattice, which limits the in-plane modification of spins.

### TRMOKE: the dynamical signal of light-induced transient spin polarization

**Figure 4.**
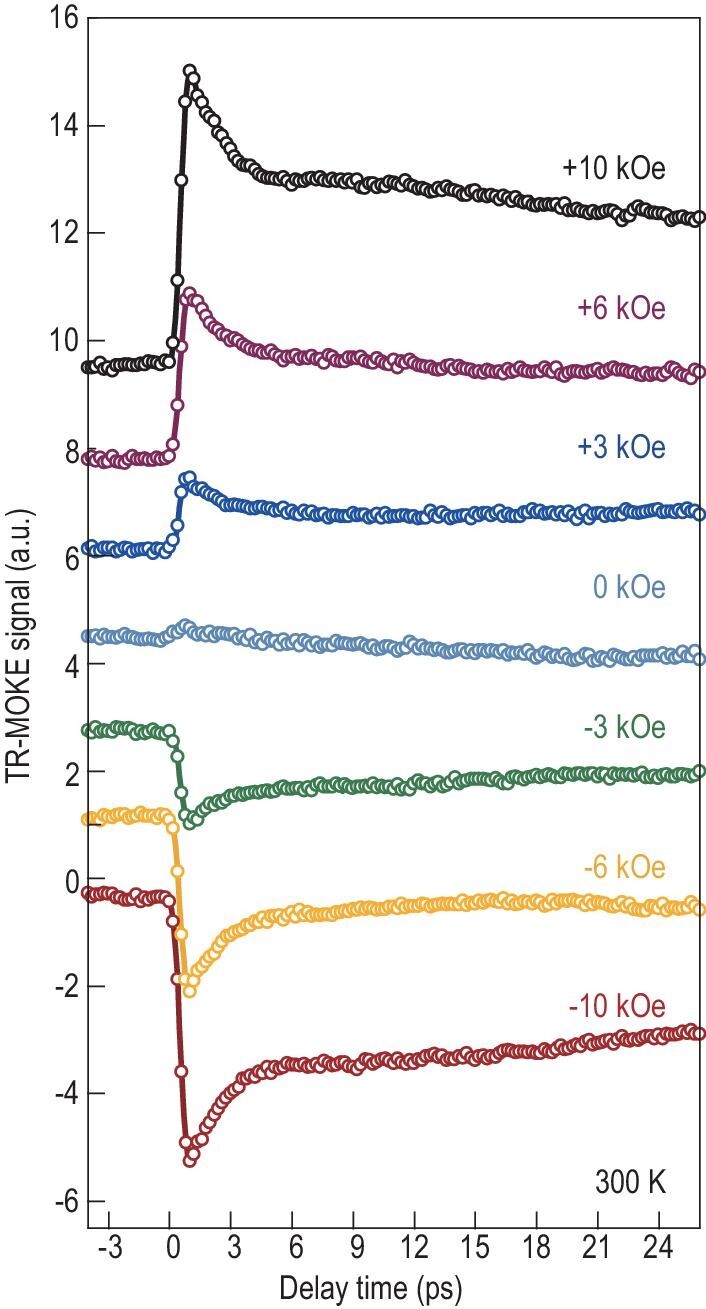
TRMOKE results of the (FGT/CS)_3_ superlattice. TRMOKE signals for the (FGT/CS)_3_ superlattice under varying magnetic fields from −10 kOe to +10 kOe. The signal deducted uncorrelated noise from polar TRMOKE, reflecting the change in the *z*-component of the magnetization, which is proportional to the change of the Kerr rotation angle.

To explore the origin of room-temperature spin currents in the (FGT/CS)_3_ superlattice, we performed TRMOKE measurements, a widely used tool that directly reflects magnetization changes during photoexcitation [9,10]. Particularly in the non-magnetic materials and initial paramagnetic state of magnets, TRMOKE techniques can reveal unconventional behavior such as transient spin polarization [4,13] or laser-induced magnetism [16,44]. A simplified block diagram of the set-up is depicted in Fig. S10, in which the (FGT/CS)_3_ superlattice was exposed to an 800-nm laser beam with a duration of 100 fs, and the out-of-plane component of magnetization was probed using a 400-nm laser beam (Section S9). Figure 4 shows TRMOKE signals (for the extraction process see Methods), which start to appear upon the arrival of the pump pulse (at the delay time t = 0 ps) and reach their peak at t = 1.0 ps. After t = 1.0 ps, the TRMOKE signals gradually relax, resembling the relaxation process observed in laser-induced demagnetization. By increasing the magnetic field, we observe a corresponding increase in the maximum value of the TRMOKE signal. Furthermore, reversing the polarity of the magnetic field resulted in a reversal of the TRMOKE signal. By fitting the trace with a two-exponential function convoluted with the Gaussian laser pulse, we further determine two characteristic timescales: 270 fs and 1.73 ps (Section S12).

Compared with the (FGT/CS)_3_ superlattice, no TRMOKE signal was detected in the pure FGT film under the same experimental conditions (Fig. S11), ruling out a paramagnetic response in the (FGT/CS)_3_ superlattice. Thus, we preliminarily attribute the magnetic-field-dependent signals in the superlattice to an indication that the laser causes transient spin polarization [4] in FGT, which stems from strengthened interfacial exchange coupling at the FGT/CS interface. The characteristic time of 270 fs corresponds to the spin polarization excitation induced by the pumping laser pulses, followed by a relaxation process of 1.73 ps. This finding aligns with the duration of the THz emission waveform.

### Theoretical analysis of the laser-enhanced proximity effect

**Figure 5.**
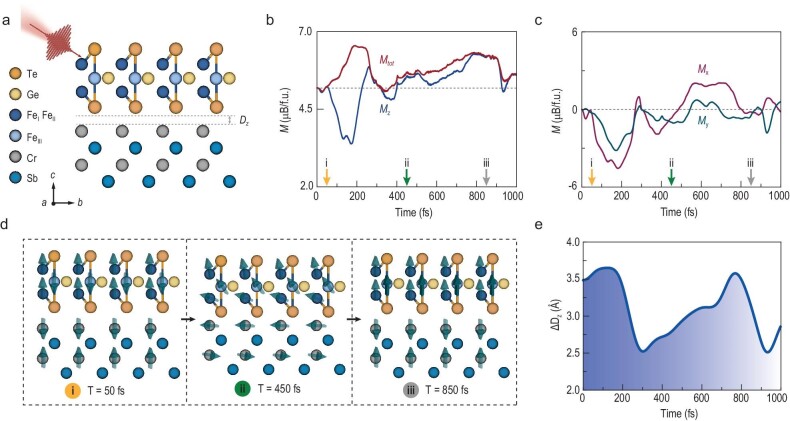
The transient spin polarization from simulations. (a) Crystal structure of the FGT/CS heterostructure with a single magnetic domain. (b, c) The transient magnetization dynamics (total magnetization in the red curve; *x*-, *y*- and *z*-direction magnetization in purple, green and blue curves) in the primitive unit cell of the (FGT/CS)_3_ superlattice. The three arrows correspond to the three moments of (i) 50 fs, (ii) 450 fs and (iii) 850 fs, respectively. (d) The local magnetic moment of magnetic atoms at the three chosen moments. (e) The temporal variation of interlayer displacement ${\mathrm{\Delta }}{{{\mathrm{D}}}_{\mathrm{z}}}$ upon photoexcitation during the same process in (b, c).

To clarify the underlying mechanisms driving the ultrafast spin dynamics observed above the *T*_C_, we conducted corresponding theoretical simulations. Figure 5a illustrates the construction of the FGT/CS heterostructure with a single magnetic domain, mimicking the experimental superlattice structure. In this study, a pump light with a wavelength of 800 nm and a fluence of 0.12 mJ/cm² was selected to replicate the optical excitation process observed in experiments. Figure 5b and c depicts the increase in the overall magnetic moment after photoexcitation, consistent with the results of TRMOKE and THz emission. Under illumination, the total magnetic moment tilts from the *z*-axis to the *x-y* plane, as evidenced by during-pulse demagnetization (Fig. S11). Following the disappearance of laser pulses, the magnetic moment reorients towards the *z*-axis, resulting in a sudden surge in magnetization, as illustrated in Fig. 5b and c. To better comprehend the dynamical involvement of individual magnetic atoms, we analyzed specific moments (50 fs, 450 fs and 850 fs) indicated by arrows in Fig. 5b and c. Figure 5d illustrates the orientation of the local magnetic moment at these moments. At the moment of 50 fs, in the absence of light, the magnetic moment of the Fe_III_ atom in FGT material exhibits an antiparallel orientation with the Fe_I_ and Fe_II_ atoms, indicating the antiferromagnetic coupling between the Fe_III_ and Fe_I_ (or Fe_II_) atoms. Antiferromagnetic coupling is also observed between Cr atoms in CS and at the FGT/CS interfaces. The antiferromagnetic coupling direction changes from the *z*-axis to the *x-y* plane at 450 fs. Notably, the exchange coupling between Fe_III_ and Fe_I_ (or Fe_II_) changed from antiferromagnetic to ferromagnetic coupling, leading to changes in magnetic configuration between 50 fs and 850 fs.

Moreover, a significant (up to 1 Å) relative interlayer displacement between the FGT and CS layers (Fig. 5e) was observed, defined as the *z*-direction displacement between Te atoms and Cr atoms in Fig. 5a. We investigated three cases to confirm its impact on laser-driven enhanced magnetization: no fixed atoms, atoms with only the *z*-direction motions, and fixed atoms. Figure S14 results indicate that the total magnetic moment increases only when atomic motion [45,46] in the *z*-direction is allowed.

Figure S15 demonstrates that ultrafast lasers open an effective channel to change the potential energy surface in a non-thermal way, enabling the system to switch to the metastable magnetic state with enhanced magnetization. These results stem from a huge interlayer displacement and enhanced magnetic exchange interactions between the Fe_III_ atom and Fe_I_ (or Fe_II_) atoms induced by the femtosecond laser in the FGT/CS heterostructure.

## DISCUSSION

At first glance, our observation of spintronic THz emission in the (FGT/CS)_3_ superlattice at room temperature may be counterintuitive, given the disappearance of ferromagnetic order in the superlattice above 206 K. However, the femtosecond optical pulses substantially excite the spin polarization in a non-equilibrium state, thus allowing the generation of ultrafast spin currents. The collective experimental and theoretical observations encapsulate the entire process as follows.

The absorption of the 800-nm pump laser by the (FGT/CS)_3_ superlattice results in the shortening of the interlayer distance between the FGT and CS layer in just a few hundred femtoseconds. This, in turn, amplifies the proximity effect or the interaction between the two materials sufficiently to cause the spin polarization of FGT above *T*_C_. Meanwhile, the magnetic moment of CS reorients from out-of-plane to in-plane, polarizing the spin of FGT in the in-plane direction. Here, the spin polarization along the specific in-plane direction is a consequence of the unique electronic structure and spin-orbit coupling interactions present. The underlying crystal lattice and the associated spin textures allow for a preferential alignment of spins due to the influence of spin-orbit coupling interactions. These interactions can lead to a non-uniform spin distribution, which favors alignment in a particular direction. Furthermore, the asymmetry at the interface may also result in the breaking of spin degeneracy, thus enhancing the probability of spin polarization along a specific orientation rather than resulting in a completely non-polarized state. Because the pump photon energy of 1.55 eV surpasses the optical gaps of the FGT and CS layers, photocarriers are simultaneously excited and polarized along the in-plane direction. The resulting spin-polarized current is subsequently injected into the CS layer and converted into the charge current through the spin-to-charge conversion effect, emitting THz radiation. During this process, the transient spin polarization response is captured by the TRMOKE technique.

As we discussed, the electron–electron exchange interactions at the interface dominates the laser-induced spin dynamics. We speculate that this laser-enhanced proximity effect may further induce ferromagnetic order in FGT. Nevertheless, regardless of the circumstances, our results indicate that these processes occur more rapidly than the corresponding dynamics of laser-induced demagnetization in ferromagnetic FGT [47].

## CONCLUSION

In summary, we have experimentally demonstrated the generation of above-*T*_C_ ultrafast spin currents in a 2D van der Waals (FGT/CS)_3_ superlattice, detected by emitted electromagnetic transients. Real-time first-principles simulations of the excited state dynamics post-photoexcitation identify that the key to such intriguing spin dynamics is a reduction in interlayer displacement and an enhancement of light-modulated electron–electron exchange interactions. The TRMOKE technique has provided complementary insights, unveiling optically excited instantaneous spin polarization in the (FGT/CS)_3_ superlattice. We collectively refer to these two observations as a ‘laser-enhanced proximity effect’, which holds the potential to induce transient ferromagnetism in FGT. We believe the transient THz spin dynamics above *T*_C_ revealed in this work will advance high-speed optoelectronic device applications based on 2D magnetic materials.

## METHODS

### Laser THz emission spectroscopy

In the THz emission spectroscopy system (Fig. S2a), a Ti: sapphire laser oscillator with an 800 nm central wavelength, 100 fs pulse duration and 80 MHz repetition rate was utilized. The average power of the pump pulses ranges from 10 to 150 mW, with a spot diameter of 2 mm. Two 90° off-axis parabolic (OAP) mirrors collimated and then focused the generated THz pulses onto a low-temperature-grown GaAs antenna (mounted to a silicon lens) for detection. The THz beam path was enclosed in a plastic box purged with dry nitrogen gas to minimize water vapor absorption, maintaining humidity below 1.5%.

### Extraction of the magnetic-field-dependent signals

We applied an external magnetic field perpendicular to the sample surface from −10 kOe to +10 kOe. The raw time-resolved magnetization dynamics under various applied fields are depicted in Fig. S12a. At 25 ps, the transient magneto-optical Kerr signal comprises different components, namely electrons, magnons (quantized states of spin waves) and phonons (quantized states of lattice waves). The amplitude increases with positive external fields, while for negative external fields, the signal is reversed and gradually decreases. Notably, the signal exhibits asymmetric changes with the magnetic field, approaching zero at 3 kOe. The relationship between the maximum signal values and magnetic fields is depicted in Fig. S12b. The signal exhibits inverse behavior, and the curve demonstrates odd symmetry. To obtain an effective magnetic signal, we add and subtract the positive and negative magnetic fields (Fig. S12c and d). This yields a magnetic component (Fig. S12c) that increases linearly with the magnetic field magnitude and a non-magnetic component (Fig. S12d) that remains constant. The gray area represents systematic deviation due to the objective lens focusing during measurements. This method effectively removes non-magnetic influences, allowing us to extract the laser-induced spin polarization signal during magnetic field changes. The reflectivity curve is illustrated in Fig. S12e.

### Theoretical calculation

Recent implementations of rt-TDDFT, available in the QUANTUM ESPRESSO package, were utilized for dynamical modeling [48–51]. Local density approximation (LDA) described the electronic exchange-correlation contribution to the total energy [52,53]. The valence electron wave functions were expanded using plane-wave basis sets with an energy cutoff of 120 Ry. Full-relativistic, norm-conserving pseudopotentials (NCPP) were employed to describe core electrons and the nuclei [54,55]. The Brillouin zone was sampled by an 11 × 11 × 1 Gamma-centered *k*-mesh. The structure was fully relaxed so that the convergence threshold on the ionic forces and the total energy satisfied 10^−9^ a.u and 10^−10^ Ry, respectively.

We apply a Gaussian-envelop laser pulse following a waveform:


(2) \begin{eqnarray*}
{\mathrm{E}}\left( {\mathrm{t}} \right)\,{\mathrm{ = }}\,{{{\mathrm{E}}}_{\mathrm{0}}}{\mathrm{cos}}( {{\mathrm{2\pi \omega t}}} ){\mathrm{exp}}\left[ {{\mathrm{ - }}{{{( {{\mathrm{t - }}{{{\mathrm{t}}}_{\mathrm{0}}}} )}}^{\mathrm{2}}}{\mathrm{/2}}{{{\mathrm{\sigma }}}^{\mathrm{2}}}} \right],
\end{eqnarray*}

where the pulse duration σ is 27.6 fs and the photon energy $h\omega $ is 1.55 eV. The laser field has a fluence of 0.12 mJ/cm² at the initial time t_0_ = 50 fs. We employed a reduced 5 × 5 × 1 *k*-mesh, and the time step is 0.145 fs for nuclei and 0.145 for electrons in our dynamical simulations.

## Supplementary Material

nwae447_Supplemental_File
