# Supplementary material for: Above-Curie-temperature ultrafast terahertz emission and spin current generation in a 2D superlattice (Fe3GeTe2/CrSb)3
Source: Natl Sci Rev. 2024 Dec 11;12(3):nwae447. doi: 10.1093/nsr/nwae447 (PMC11827587; doi:10.1093/nsr/nwae447)
Supplement: nwae447_Supplemental_File [file nwae447_supplemental_file.docx]

Supplementary Data

Above Curie Temperature Ultrafast Terahertz Emission and Spin Current Generation in a Two-Dimensional Superlattice (Fe_3_GeTe_2_/CrSb)_3_

Peiyan Li,^1,2^,† Na Wu,^3,4^,† Shanshan Liu,^5^,† Yu Cheng,^3,4^† Piming Gong,^3,4^ Junwei Tong,^6^ Jianan Liu,^3,4^ Wei He,^3,4^* Faxian Xiu,^5^* Jimin Zhao,^3,4,7^* Sheng Meng,^3,4,7^* and Xiaojun Wu^1,2,8,9^*

Correspondence to: xiaojunwu@buaa.edu.cn, smeng@iphy.ac.cn, jmzhao@iphy.ac.cn, faxian@fudan.edu.cn, hewei@iphy.ac.cn.

**Table of Contents**

[**Section 1.** Sample characterization and characterization 3](#_Toc181228818)

**[Section 2.](#_Toc181228819)** [Terahertz emission spectroscopy and the dependence of laser pump fluence, azimuth, and laser polarization direction 4](#_Toc181228819)

**[Section 3.](#_Toc181228820)** [Influence of the polarization state of the pumping pulses on the THz emission from the (FGT/CS)](#_Toc181228820)[3](#_Toc181228820) [superlattice 6](#_Toc181228820)

[**Section 4.** Exclusion of the contribution of laser-dependent components 7](#_Toc181228821)

[**Section 5.** Low-temperature THz emission measurement 9](#_Toc181228822)

[**Section 6.** Verification of the dependence of the THz signal on the pump polarization below the *T*_C_. 11](#_Toc181228823)

[**Section 7.** The design and photos of used in-plane and out-of-plane magnets 12](#_Toc181228824)

[**Section 8.** THz waveforms of the (FGT/CS)_3_ superlattice at 2000 Oe magnetic field 13](#_Toc181228825)

[**Section 9.** Time-resolved magneto-optical Kerr effect (TRMOKE) measurement 14](#_Toc181228826)

[**Section 10.** The TRMOKE signal at 0 Oe 15](#_Toc181228827)

[**Section 11.** Extracting magnetic correlation components 16](#_Toc181228828)

[**Section 12.** TRMOKE characteristic times fitting 17](#_Toc181228829)

[**Section 13.** Simulations of laser-induced magnetization dynamics in three cases 18](#_Toc181228830)

[**Section 14.** Understanding of the laser-induced exchange coupling enhancement and the interlayer displacement in the (FGT/CS)_3_ superlattice. 19](#_Toc181228831)

[**Section 15.** Exploration of the (FGT/CS)_3_ superlattice. 21](#_Toc181228832)

[**References**](#_Toc181228833)**.** 22

**Supplementary Text**

**Section 1. Sample preparation and characterization.**

Fe_3_GeTe_2_ (FGT) and CrSb (CS) belong to the space group *P6_3_/mmc* with a hexagonal crystal shape. FGT comprises five sublayers, possessing lattice constants of a = b = 3.991 Å, c = 16.396 Å, in agreement with theoretical calculations. The growth of FGT and CS was assessed using reflection high-energy electron diffraction (RHEED) to confirm a flat surface (Fig. S1a). A-type antiferromagnetic CS exhibits the NiAs phase, characterized by lattice constants of a = b = 4.108 Å, c = 5.440 Å, and the Néеl temperature up to 700 K. Chromium spins are antiferromagnetically oriented between adjacent planes and ferromagnetically aligned in-plane. A cross-sectional scanning transmission electron microscopy (STEM) image confirms the presence of sharp interfaces between the FGT and CS layers in the (Fe_3_GeTe_2_/CrSb)_3_ (abbreviated as (FGT/CS)_3_) superlattice (Fig. S1b). [1]


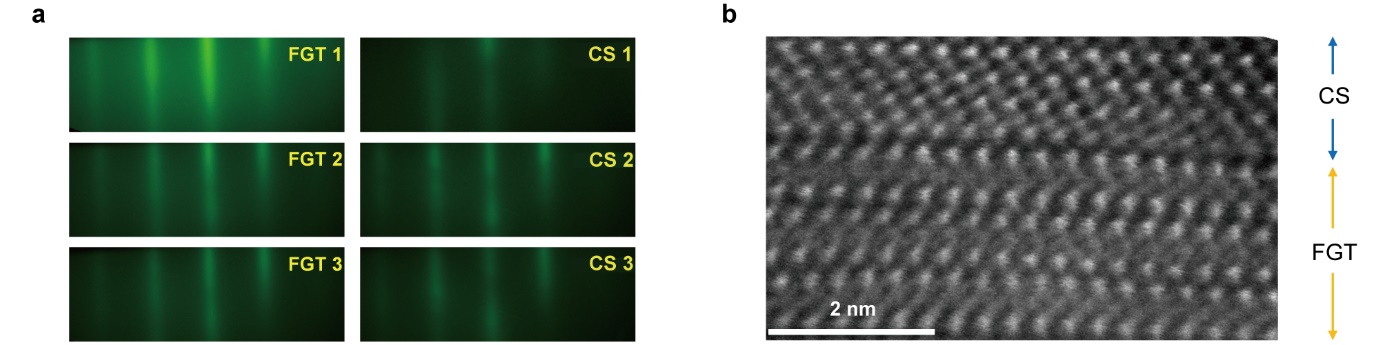


**Figure S1.** (a) RHEED pictures were taken after each FGT and CS layer growth. (b) The cross-section STEM image.

Section 2. Terahertz emission spectroscopy and the dependence of laser pump fluence, azimuth, and laser polarization direction


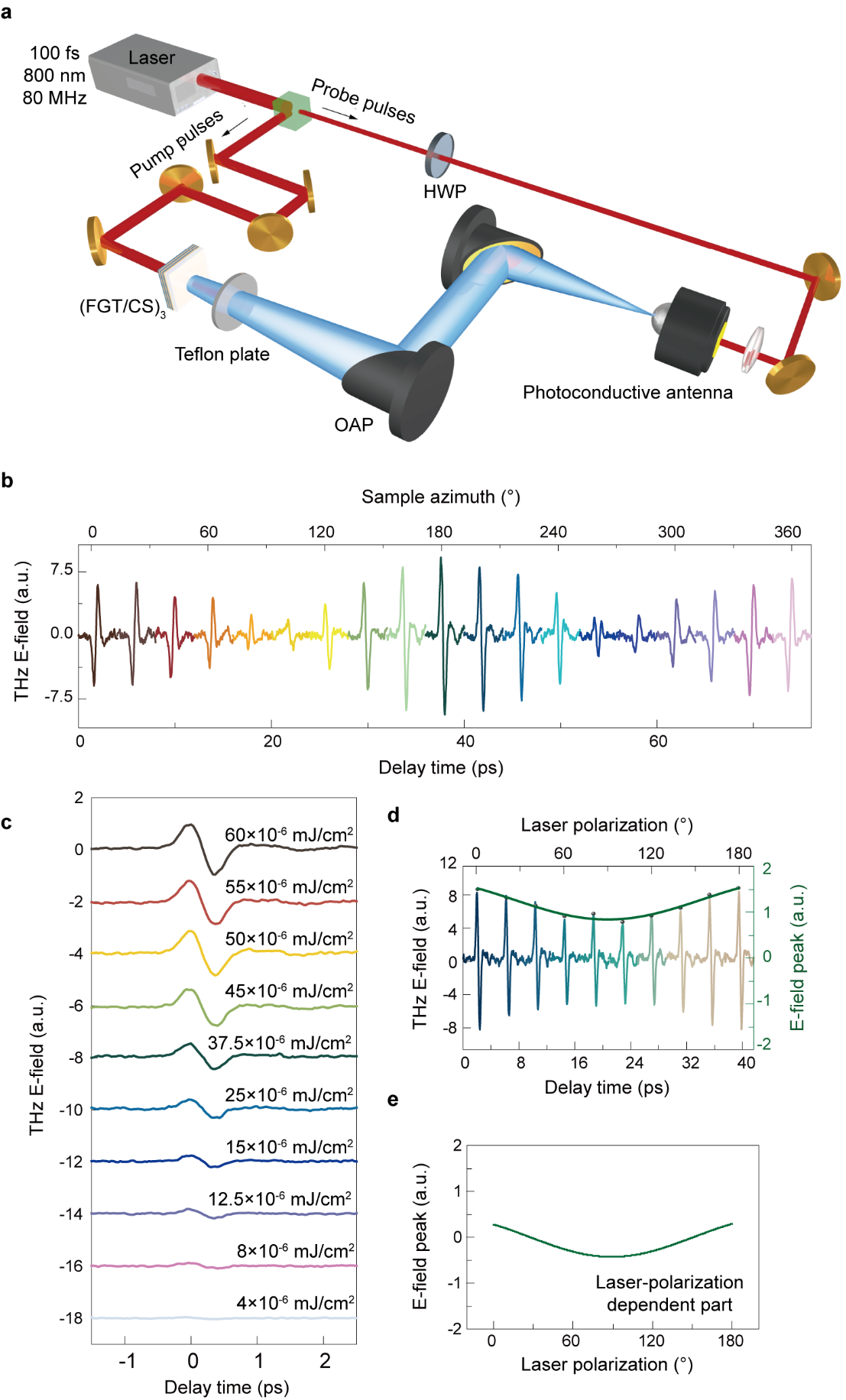


**Figure S2.** (a) Schematics of the terahertz (THz) emission spectroscopy and detection setup. [2,3] PBS: polarization beam splitter; HWP: half-wave plate; OAP: Off-axis parabolic mirror. (b) Dependence of the THz amplitude of the (FGT/CS)_3_ superlattice on azimuth angle. (c) Typical THz emission temporal waveforms of (FGT/CS)_3_ superlattice with different pump fluences. (d) The relationship between the THz amplitude of the (FGT/CS)_3_ superlattice and the laser polarization direction. (e) Only a small fraction of THz radiation in superlattice is related to the laser polarization.

Section 3. Influence of the polarization state of the pumping pulses on the THz emission from the (FGT/CS)_3_ superlattice

We measured the THz emission waveforms from the (FGT/CS)_3_ superlattice under circular and linear laser pumping, as shown in Fig. S3. The THz radiation emitted by the (FGT/CS)_3_ superlattice excited by both linear and circularly polarized laser pulses exhibits linear polarization. The intensity of THz emission is enhanced when using linearly polarized laser pulses compared to circularly polarized ones. There is no obvious difference between the waveforms obtained with right and left circularly polarized laser pumping. Circularly polarized light can be decomposed into two orthogonal components, one polarized along the *y*-axis and the other along the *x*-axis, each with identical magnitudes. We consider that each of the two orthogonal components independently contributes to the THz emission and the total amplitude of the THz emission can therefore be considered as the sum of these contributions. The THz emission from the CS layer induced by orthogonally linearly polarized laser beams shows opposite signs, resulting in the superposition of two contributions reducing the THz signal intensity of the (FGT/CS)_3_ superlattice. These results exclude the THz generation mechanism associated with circularly polarized lasers, such as the inverse Faraday effect.


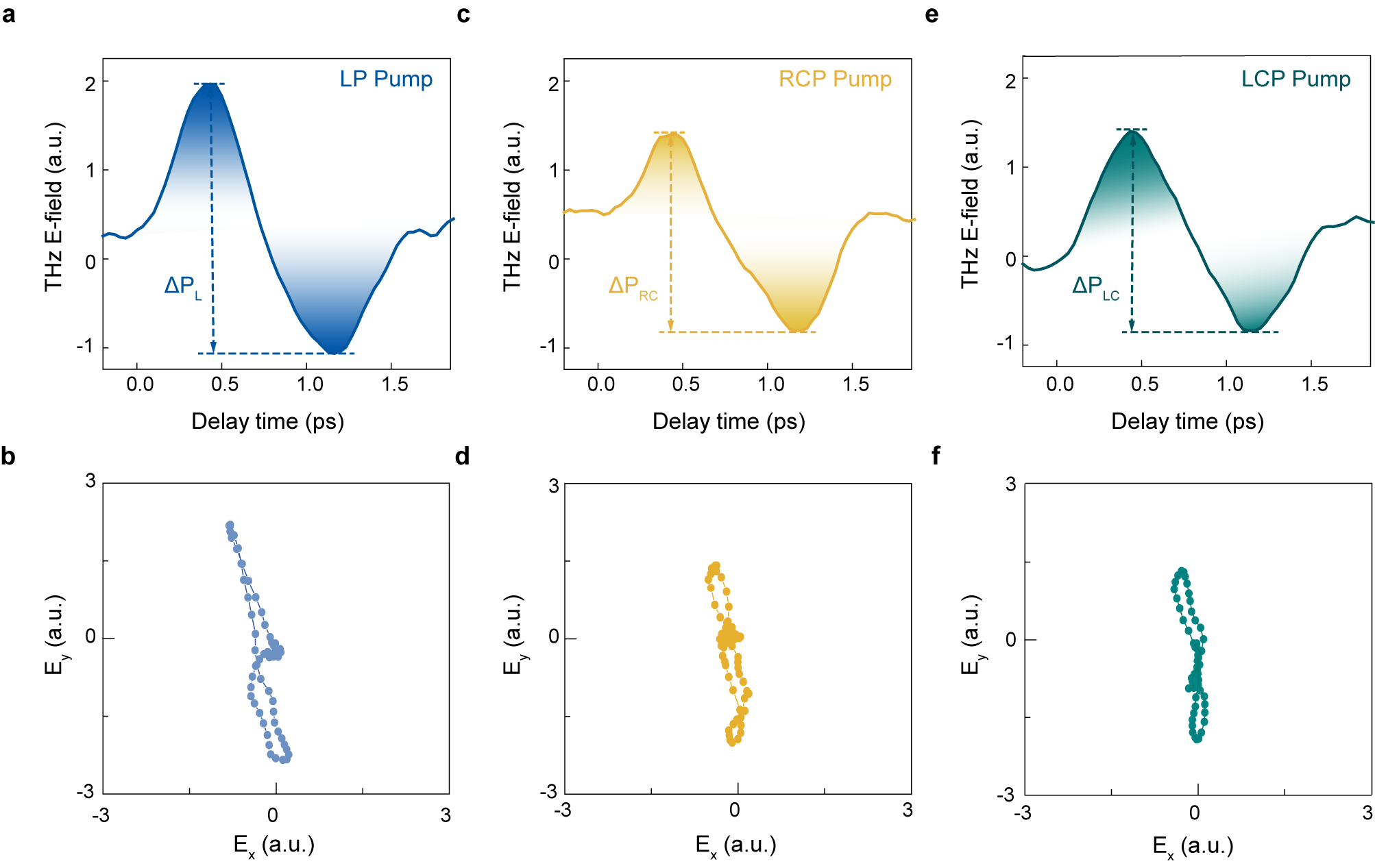


**Figure S3.** THz signals and corresponding polarization states emitted from the (FGT/CS)_3_ superlattice under excitation by (a-b) linearly polarized (LP), (c-d) right circularly polarized (RCP), and (e-f) left circularly polarized (LCP) laser pulses.

Section 4. Exclusion of the contribution of laser-dependent components

We partition the total THz radiation from the (FGT/CS)_3_ superlattice into two distinct components: the laser-polarization-dependent component $\overrightarrow{E_{\alpha}}$ and the laser-polarization-independent component $\overrightarrow{E_{\theta}}$. $\overrightarrow{E_{\theta}}$ consistently perpendicular to the spin orientation, thus stabilizing the azimuth angle of the superlattice ensures the constancy of $\overrightarrow{E_{\theta}}$. Conversely, the orientation of $\overrightarrow{E_{\alpha}}$ is contingent upon the laser polarization direction. The THz radiation emitted by the superlattice attains its maximum and minimum values by fixing the sample azimuth and changing the laser polarization direction. [2] The maximum THz electric field value, denoted as $\overrightarrow{E_{\max}}$, is expressed as $\overrightarrow{E_{\theta}}+\overrightarrow{E_{\alpha}}$, while the minimum value, $\overrightarrow{E_{\min}}$, is represented as $\overrightarrow{E_{\theta}}-\overrightarrow{E_{\alpha}}$. Therefore, the $\overrightarrow{E_{\alpha}}$ and $\overrightarrow{E_{\theta}}$ can be derived as:

$$\overrightarrow{E_{\alpha}}=\frac{\overrightarrow{E_{\max}}-\overrightarrow{E_{\max}}}{2}$$

$$\overrightarrow{E_{\theta}}=\frac{\overrightarrow{E_{\max}}+\overrightarrow{E_{\max}}}{2}$$

That is, by varying the pump polarization affecting the THz signal, the laser-polarization-independent component corresponding to the THz electric field vector can be accurately extracted with the time-domain waveform depicted in Fig. S4.


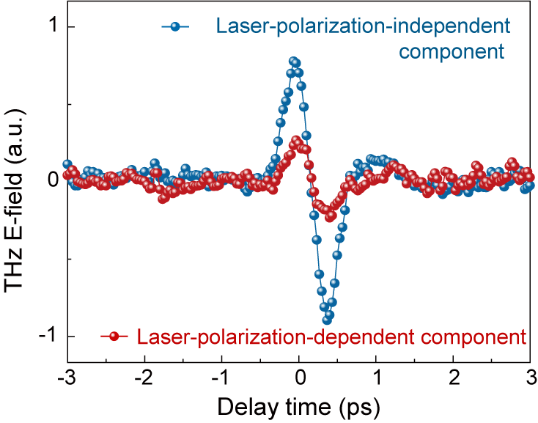


**Figure S4.** Extraction of THz waveforms from both the laser-polarization-independent and laser-polarization-dependent components.

The laser-polarization-dependent component corresponds to the THz signal from the CS layer. The CS with NiAs phase is a semiconductor with inherently low conductivity, whose THz emission mechanism is related to second-order nonlinear effects and photo-induced carriers. The incident photons, through the process of difference-frequency generation—a second-order nonlinear phenomenon—can induce a low-frequency magnetic field [4], resulting in a THz signal that is highly sensitive to the laser polarization (Fig. S2e). Furthermore, when the incident photon energy surpasses the bandgap of materials, photogenerated carriers are accelerated under intrinsic electric fields, thereby facilitating additional THz radiation [5]. Photocurrent effects, such as the surface depletion field, photo-Dember effect, and photothermal effect, also play significant roles in the THz emission process. However, it is important to note that while the photon drag effect (PDE) is a well-known contributor to THz generation under intense light beams with high photon flux, its influence in our experiments is negligible. The generation of PDE photocurrents requires photons with an oblique incidence to have a finite in-plane momentum that can be transferred to electrons along the momentum transfer direction to generate currents.

Section 5. Low-temperature THz emission measurement

**The experimental investigations at low temperatures employed a cryostat filled with liquid nitrogen, as depicted in Fig. S5a. Figure S5b-c shows the raw THz radiation waveforms and the corresponding Fourier transform results emitted from the (FGT/CS)_3_ superlattice at temperatures ranging from 80 K to 300 K.** We observe a significant transition in the emitted raw THz signals as the temperature increases from 80 K to 300 K, with 200 K serving as a critical point: Below 200 K, the THz amplitude remains relatively stable, but beyond it, a distinct decline occurs, followed by stabilization beyond 200 K. This transition indicates the presence of a distinct mechanism for spin-current generation above the Curie temperature (*T*_C_), differing from the laser-induced ultrafast demagnetization observed below *T*_C_. **The datasets underwent ten rounds of averaging under constant temperature conditions.**


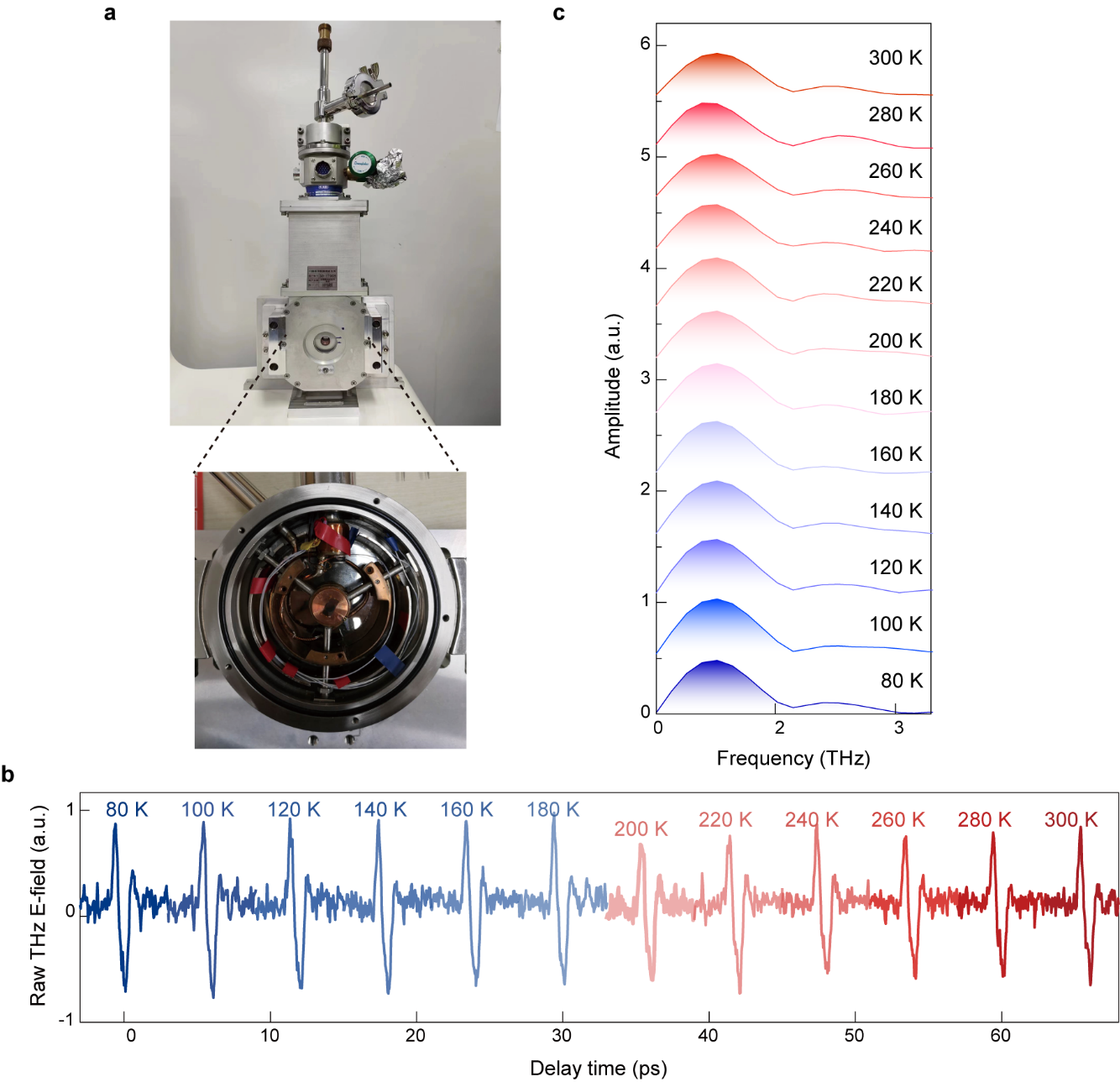


**Figure S5.** (a) Photographs of the overall and internal structure of liquid nitrogen cryostat. (b-c) Raw temporal waveforms and Fourier-transferred spectra as a function of the temperature. These signals were horizontally offset for clarity.

To accurately delineate the relationship between the intensity of THz radiation and temperature, we further individually projected each raw THz time-domain signal onto two reference signals with different pump fluences (Fig. S6), respectively. Specifically, we conducted an inner product operation at each temperature point between the raw THz signal and each reference signal. Then we averaged the projections from the two reference signals and depicted the outcomes in Fig. 3b. These resulting projection outcomes enable the quantification of fluctuations in THz signal strength across various temperatures, with changes in projection values indicating corresponding variations in the intensity of THz radiation relative to temperature changes.


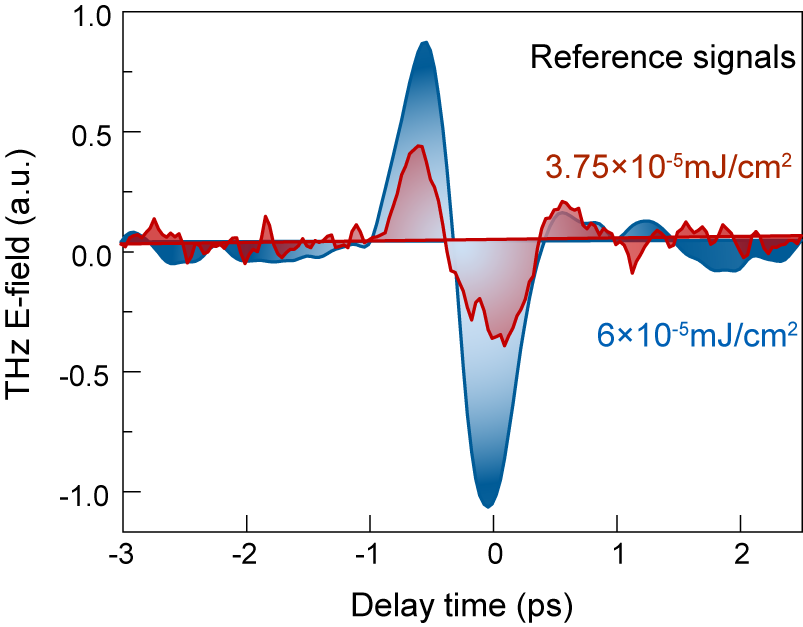


**Figure S6.** The reference THz signals are used for signal projection. The enhanced signal-to-noise ratio achieved through signal projection enabled the accurate extraction of meaningful information from noisy THz signals.

Section 6. Verification of the dependence of the THz signal on the pump polarization below the *T*_C_.

**By rotating the half-wave plate, the relationship between THz radiation and laser polarization below and above *T*_C_ is illustrated in Fig S7. These results** reveal a remarkable consistency in the overall trends of THz polarity dependence on pump polarization, both at 100 K and 300 K. However, a difference emerges in the laser-independent THz component, which is related to the spin-to-charge conversion effect mechanism, specifically, this component is weaker at 300 K compared to 100 K. Based on the invariant conversion efficiency, we attribute this result to the different spin current origins below and above *T*_C_ in the (FGT/CS)_3_ superlattice. Below *T*_C_, the spin current is related to ultrafast demagnetization processes, whereas above *T*_C_, it is attributed to the laser-enhanced magnetic proximity effect.


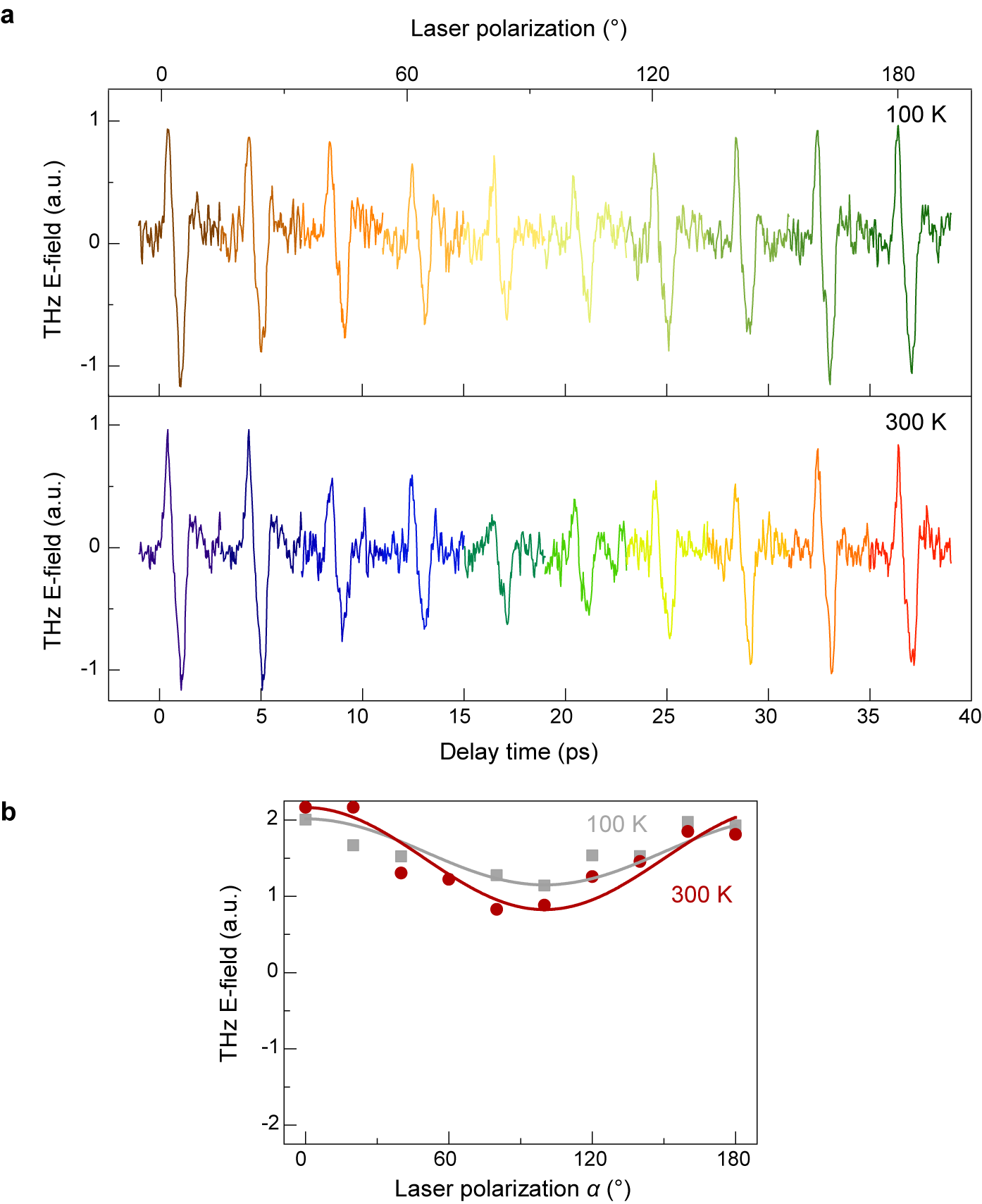


**Figure S7.** (a) Comparison of the THz signal of the (FGT/CS)_3_ superlattice at 100 K and 300 K. (b) Corresponding THz amplitude as a function of laser polarization at 100 K and 300 K.

Section 7. The design and photos of used in-plane and out-of-plane magnets

**Figure S8a depicts an electromagnet used in the experiment to generate an in-plane magnetic field with a maximum strength of 2000 Oe. At 300 K, an annular magnet was utilized to apply a 2000 Oe out-of-plane magnetic field as illustrated in Fig. S8b. At 100 K, a** three-dimensional (3D)**-printed bracket (Fig.S8c) is used to install the annular magnet (2000 Oe) outside the low-temperature thermostat to apply an out-of-plane magnet. Due to the constraints posed by the cryostat cavity, however, the magnetic field strength applied to the superlattice was reduced to 400 Oe.**


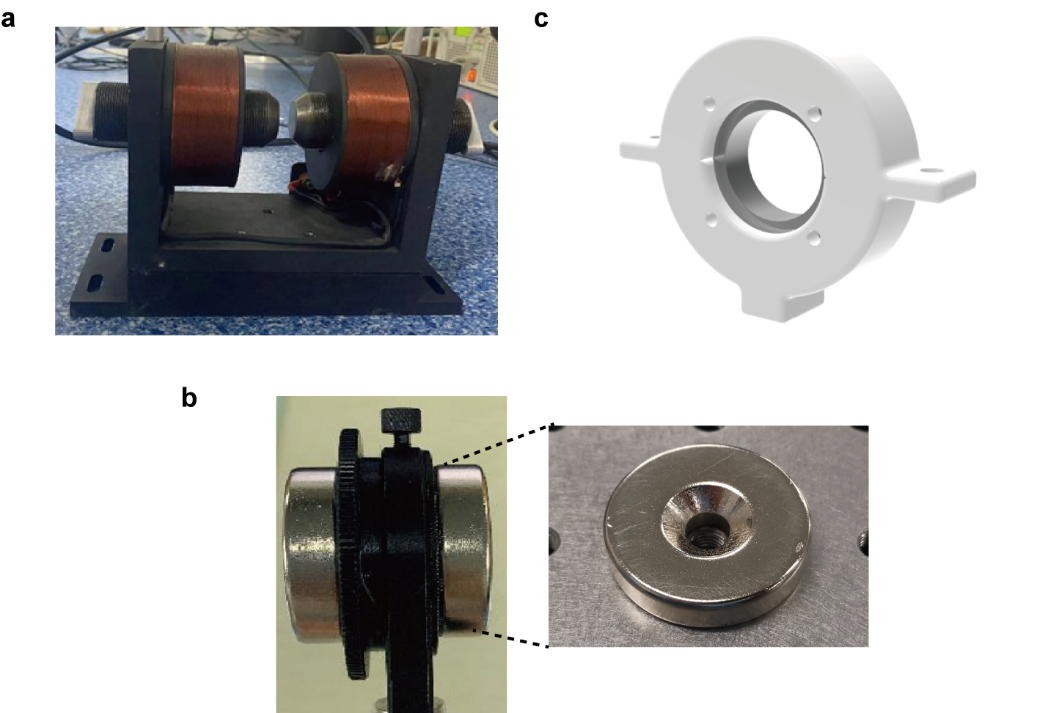


**Figure S8.** Photographs of in-plane and out-of-plane magnetic fields utilized in THz emission experiments at room and low temperatures. (a-b) The photographs of an electromagnet employed for applying an in-plane magnetic field and a circular magnet used to apply an out-of-plane magnetic field at room temperature. (c) The 3D-printed out-of-plane magnet holder facilitates the installation of the annular magnet outside the cryostat.

Section 8. THz waveforms of the (FGT/CS)_3_ superlattice at 2000 Oe magnetic field


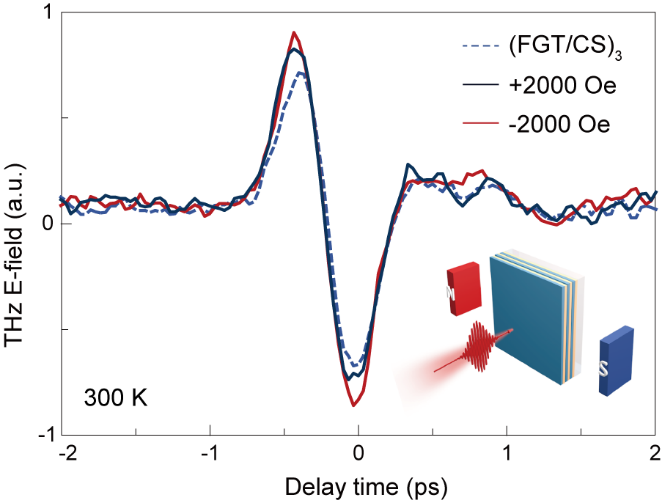


**Figure S9.** Emitted THz electric field waveforms from the (FGT/CS)_3_ superlattice under the in-plane magnetic fields. The THz amplitude slightly increases when subjected to external magnetic fields of ±2000 Oe aligned parallel to the sample plane.

Section 9. Time-resolved magneto-optical Kerr effect (TRMOKE) measurement

**In the TRMOKE system, [6]** **t**he 800-nm center wavelength and 100-fs pulse duration pulse train are produced by a Ti: sapphire oscillator operating at a repetition rate of 5.2 MHz. The laser beam is split by a polarizing beam splitter and directed through a BBO crystal with a thickness of 200 μm, resulting in the generation of 800 nm pump and 400 nm probe beams with mutually perpendicular polarization. The temporal separation between pump excitation and probe detection is achieved by manipulating the optical path of the pump beam using a delay stage. Focusing the pump and probe beams through a 20 x objective lens with a numerical aperture (NA) of 0.5 and normal axis incidence (*z*-axis) onto the superlattice produces a beam spot size of several tens of microns (1/e^2^ radius). The detection beam, upon reflection, passes through a photoelastic modulator (PEM) and is subsequently separated into two mutually perpendicular polarization pathways using a Wollaston prism (WP). It is noteworthy that the detection geometry is solely sensitive to the out-of-plane component of the magnetization, $\vec{\text{M}_{\text{z}}}$.


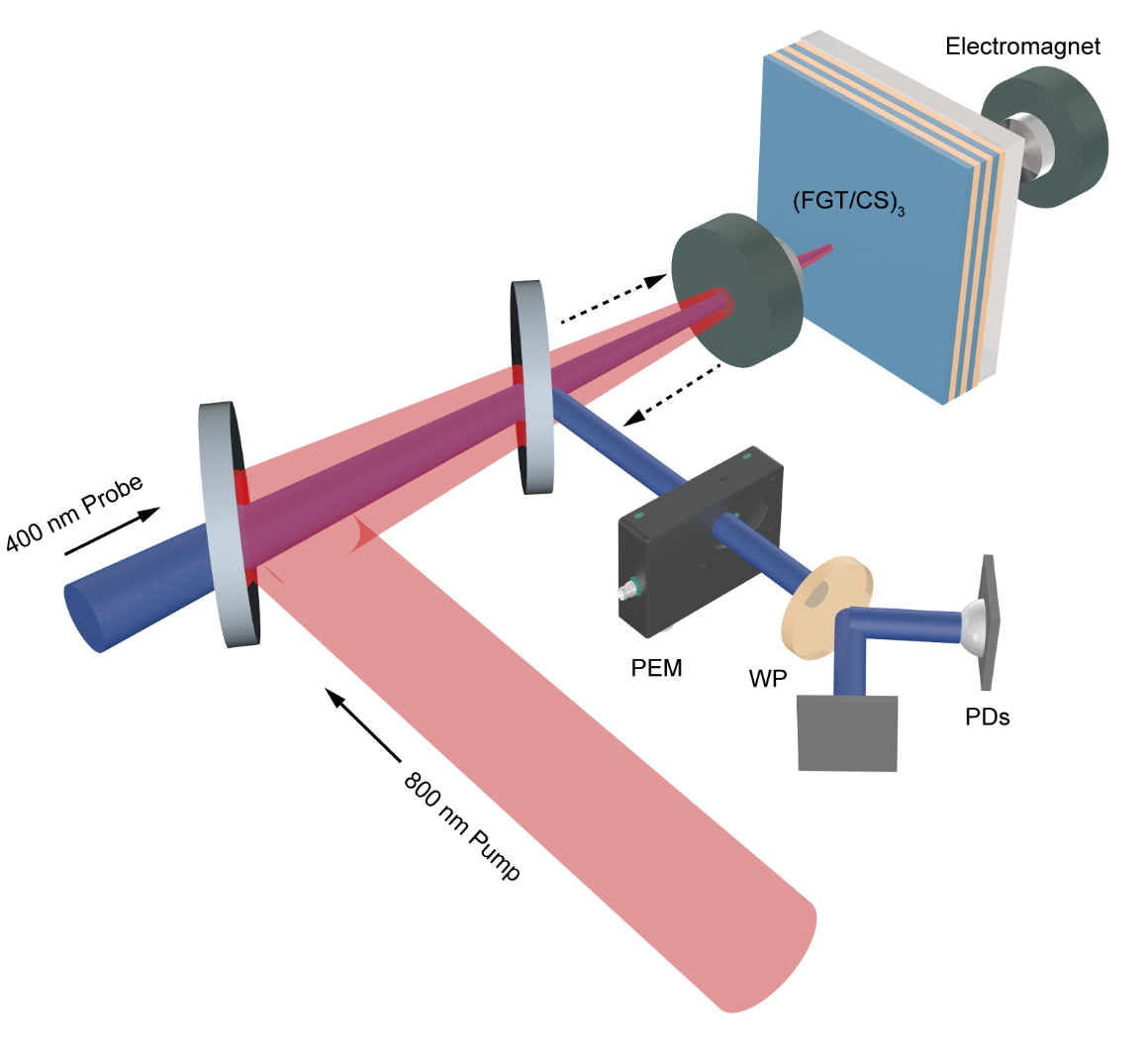


**Figure S10.** Schematic geometry of the TRMOKE system.

Section 10. The TRMOKE signal at 0 Oe

At an external magnetic field strength of 0 Oe, the principal spin polarization induced by femtosecond laser excitation aligns predominantly in the in-plane direction, as evidenced by THz emission results. Conversely, the TRMOKE detectors are solely sensitive to external components, hence only capturing weak spin polarization contributions (Fig. S11a).

Additionally, to prove that the TRMOKE curve we obtained does not belong to paramagnetic signals, we checked a 10 nm thick FGT with *T*_C_ of 140 K as a reference sample using the same TRMOKE measurement conditions. As depicted in Fig. S11b-c, the 10 nm thick FGT remains paramagnetic at room temperature and cannot generate detectable signals.


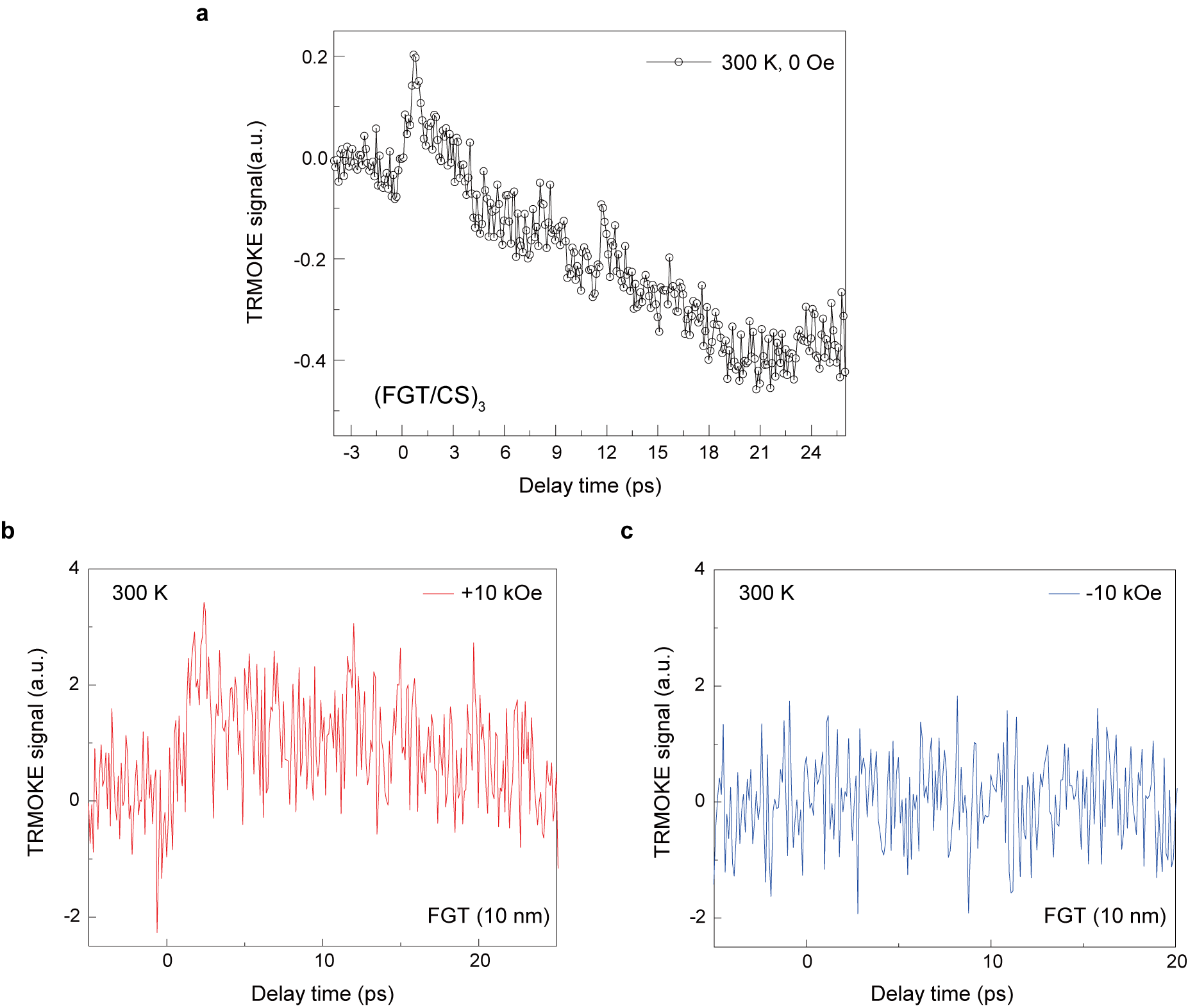


**Figure S11.** (a) The TRMOKE signal peaks on the sub-picosecond timescale before exhibiting a notable decrease. (b-c) At ±10 kOe, the TRMOKE signal of FGT disappears.

Section 11. Extracting magnetic correlation components


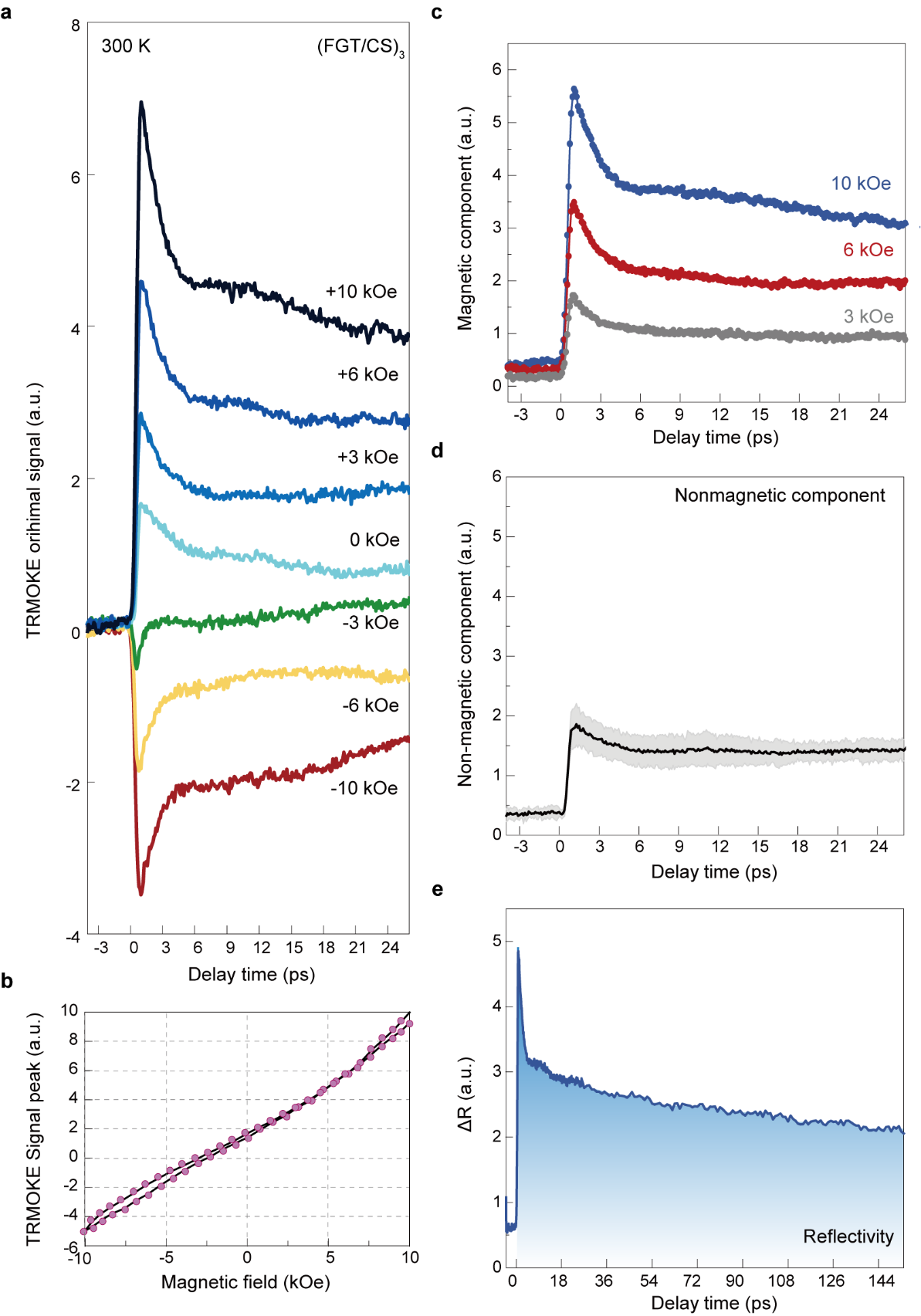


**Figure S12.** (a) The TRMOKE raw signals for the superlattice under varying external magnetic fields. (b) The transient magneto-optical Kerr signal peak curve. (c-d) Extracting magnetic and non-magnetic components. The former entails subtracting the negative magnetic field from the positive magnetic field, whereas the latter involves adding the positive and negative magnetic fields together. (e) The reflectivity curve.

Section 12. TRMOKE characteristic times fitting

The TRMOKE trace was fitted using a two-exponential function convoluted with the Gaussian laser pulse while also accounting for the influence of thermal diffusion. [7] We use the following formula to fit, and the fitting curve is depicted in Fig. S13.

$$\text{f(x)=}\left[Ae^{-\frac{t-t_{0}}{\tau_{1}}}(1-e^{-\frac{t-t_{0}}{\tau_{2}}})+\alpha_{k}(1+\frac{t}{\tau_{k}})^{-\frac{1}{2}}\right]*\Gamma(t)$$

$$\Gamma(t)=\frac{1}{\sqrt{\pi\Sigma}}e^{-\frac{\omega^{2}}{\Sigma}}$$

where the constant A is the intensity of two-exponential-function, $*$ represents the convolution product, and $\Gamma(t)$ is the Gaussian pulse representing the pump and probe pulse (full width at half maxima $\omega$ is 100 fs). The constant $\alpha_{k}$ is proportional to thermal diffusion, while $\Sigma=2\sigma^{2}=\frac{\omega^{2}}{4\ln2}\approx0.36067376\times\omega^{2}$. The time constants, $\tau_{1}$ and $\tau_{2}$, obtained in our experiment represent the excitation of spin polarization induced by the pumping laser pulses and the subsequent relaxation process. The final fitted parameters are shown in Tab. S1.


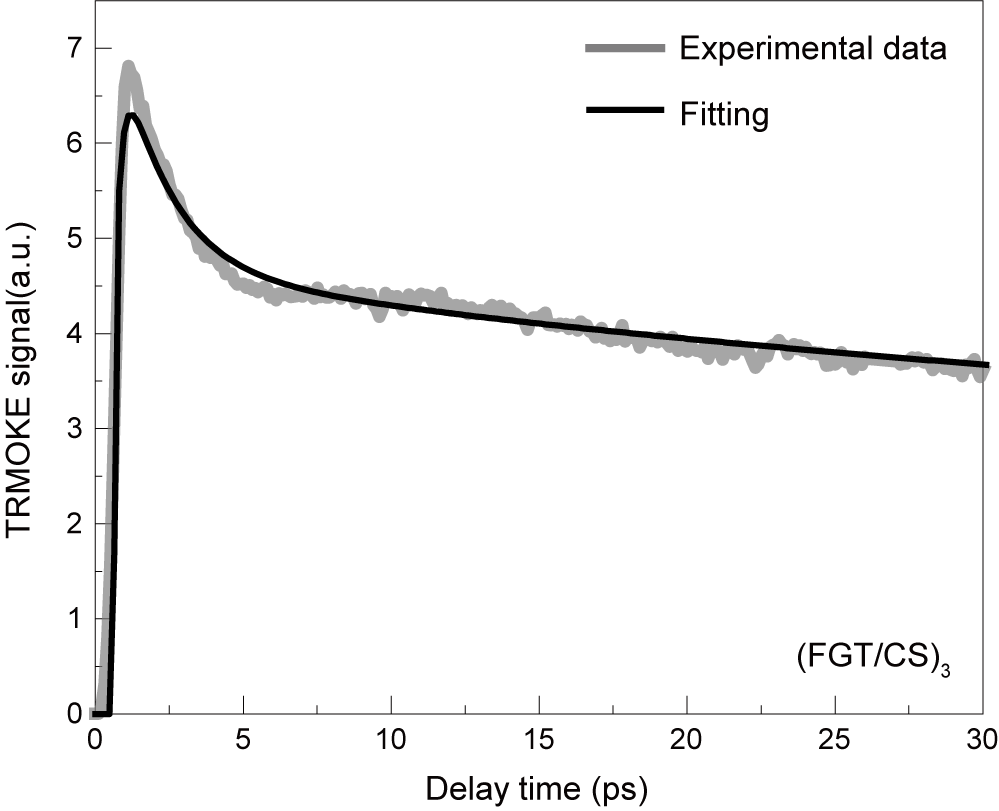


**Figure S13.** The TRMOKE fitting curve at 10 kOe. The grey and red lines correspond to the raw signal and the fitting result by a two-exponential function.

| A (a.u.) | t_0_ (ps) | $\tau_{1}$ (ps) | $\tau_{2}$ (ps) | $\alpha_{K}$ (a.u.) | $\tau_{K}$ (ps) |
| --- | --- | --- | --- | --- | --- |
| 2.599 | 0.674 | 1.706 | 0.266 | 1.806 | 46.331 |

**Table S1.** Fitting parameters.

Section 13. Simulations of laser-induced magnetization dynamics in three cases


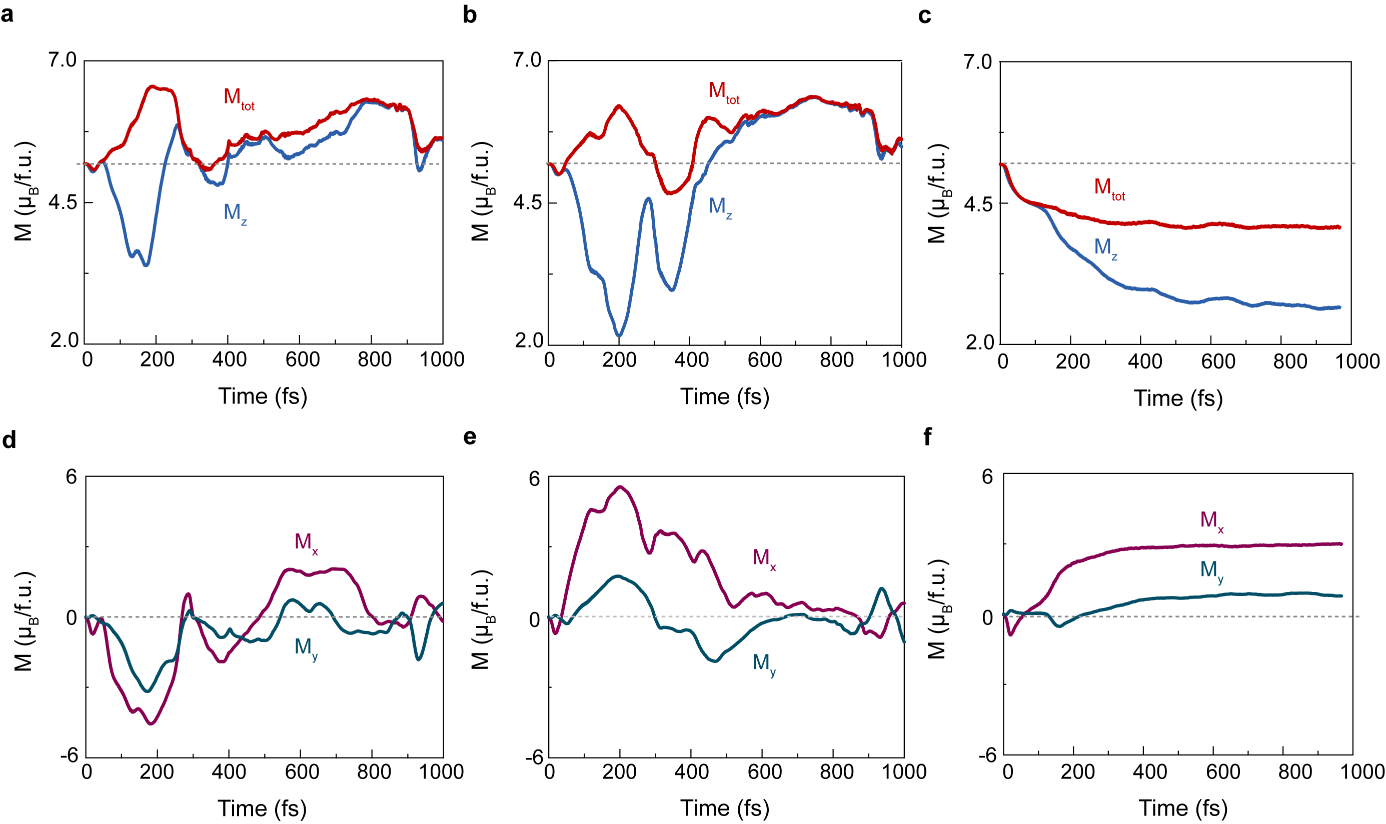


**Figure S14.** The temporal total magnetization and *x*, *y*, *z* direction magnetization. (a) and (d) show the magnetization dynamics when the atoms are not fixed. (b) and (e) demonstrate the magnetization dynamics when the atoms are allowed to move along the *z*-direction. (c) and (f) display the magnetization dynamics when the atoms are fixed. The total and *z*-direction magnetization are shown in red and blue colors, respectively. The *x*- and *y*-direction magnetization are shown in purple and green color, respectively.

Section 14. Understanding of the laser-induced exchange coupling enhancement and the interlayer displacement in the (FGT/CS)_3_ superlattice.

In laser-driven superlattice, the exchange coupling can be transiently enhanced due to the laser-induced modifications in the electronic structure, changes in magnetic anisotropy, and the interaction among spin, charge, orbital, and phonon degree of freedom. For example, the energy delivered by laser can excite electrons to higher energy states, altering the exchange interactions between localized magnetic moments. This can lead to a transient increase in the exchange coupling strength.

For the interlayer displacement we observed, the primary driver for this reduction is the light-induced creation of a nonequilibrium electron distribution within the system. This nonequilibrium electron distribution subsequently drives the generation of incoherent phonons. The excitation mechanism is the Displacive Excitation of Coherent Phonons (DECP) [8]. After generating coherent phonons, phonon-phonon interactions occur, which macroscopically drive the reduction of the in-plane interlayer distance.


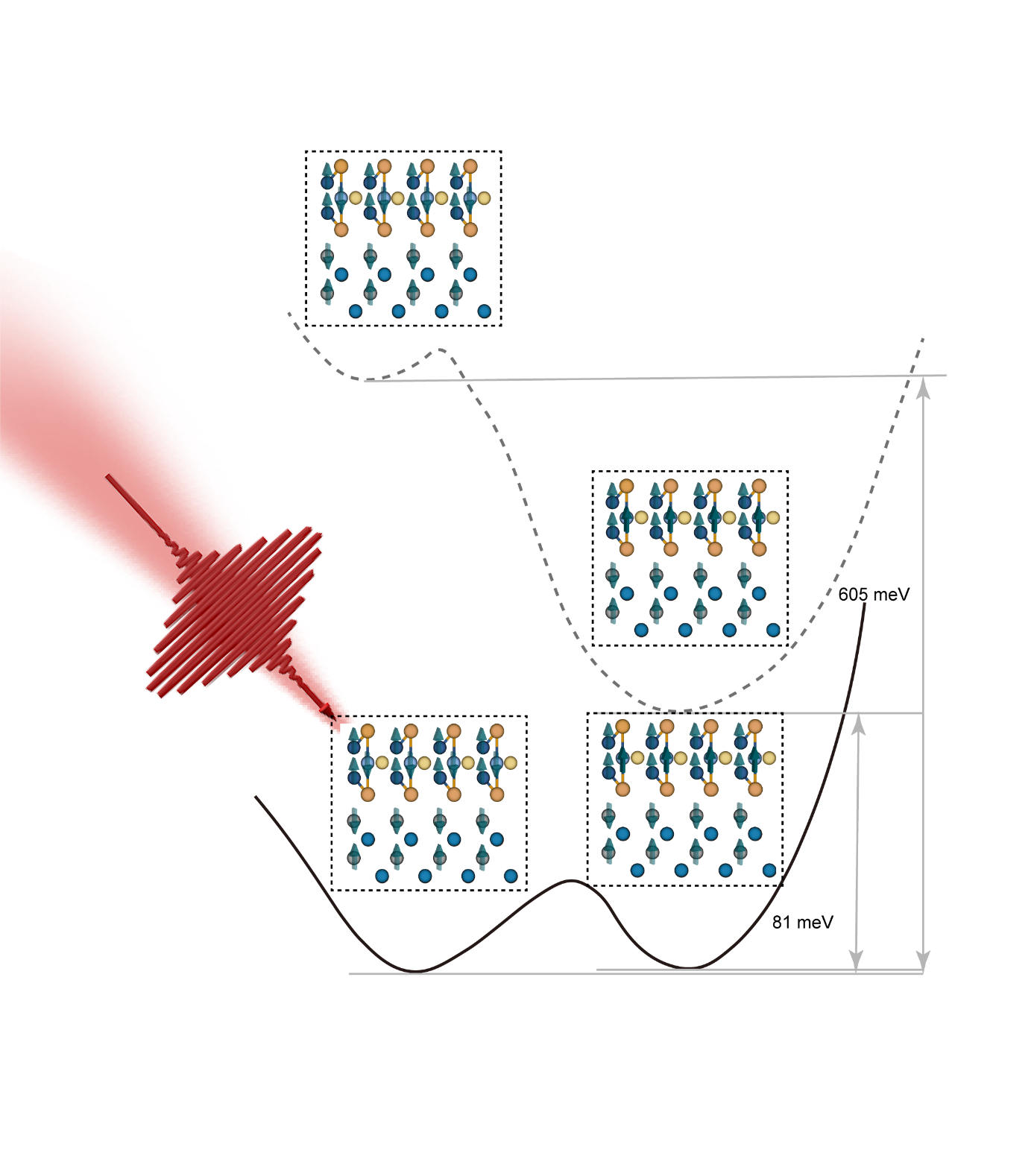


**Figure S15.** Schematic diagram of the photoinduced potential energy surface.

Section 15. Exploration of the (FGT/CS)_3_ superlattice.

Before photoexcitation, in the (FGT/CS)_3_ superlattice, the interfacial proximity effect enhances the *T*_C_ of the 4-layer FGT from 140 K to 206 K [1]. Moreover, our work demonstrates that femtosecond laser pulses cause transient spin polarization in the (FGT/CS)_3_ superlattice, leading to the generation of above-*T*_C_ spin THz currents at room temperature. The entire process is depicted in Fig. S16.


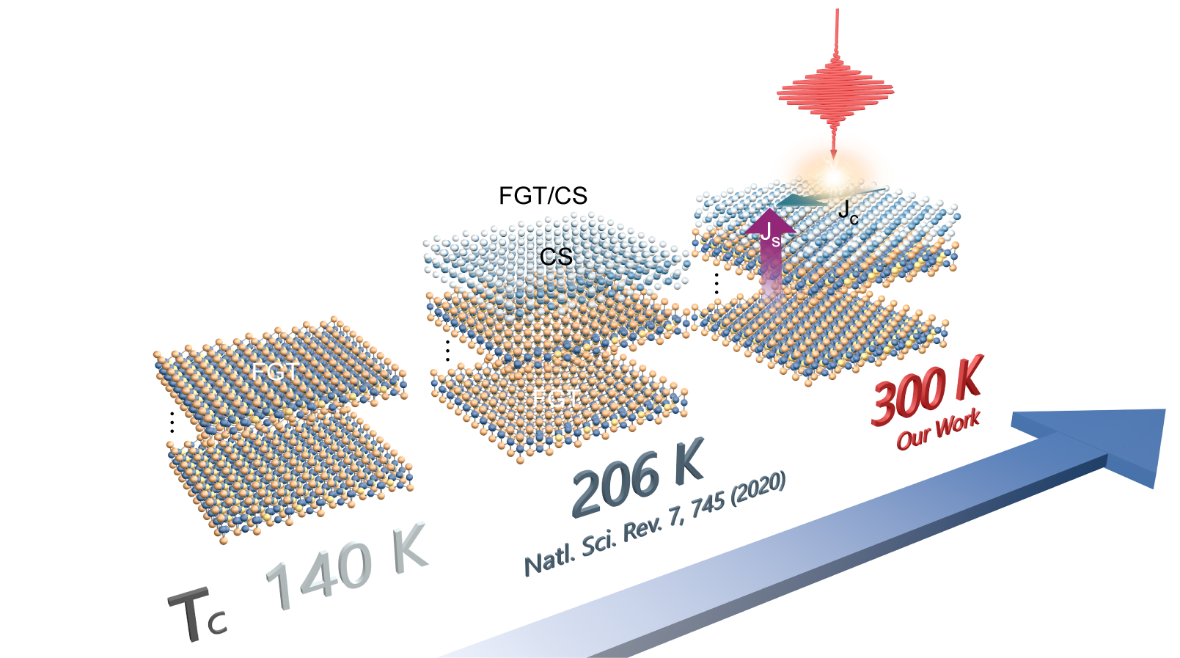


**Figure S16.** Schematic representation of the key processes in the (FGT/CS)_3_ superlattice.

References

1. Liu S, Yang K, Liu W *et al*. Two-dimensional ferromagnetic superlattices. *Natl. Sci. Rev.* 2020; **7**: 745-54.

2. Chen X, Wang H, Liu H *et al*. Generation and control of terahertz spin currents in topology-induced 2D ferromagnetic Fe_3_GeTe_2_|Bi_2_Te_3_ heterostructures. *Adv. Mater.* 2022; **34**: 2106172.

3. Wu X, Wang H, Liu H *et al*. Antiferromagnetic-ferromagnetic heterostructure-based field-free terahertz emitters. *Adv. Mater.* 2022; **34**: 2204373.

4. Qiu H, Zhou L, Zhang C *et al*. Ultrafast spin current generated from an antiferromagnet. *Nat. Phys.* 2024; **17**: 388-94.

5. Huang Y, Yao Z, He C *et al*. Terahertz surface and interface emission spectroscopy for advanced materials. *J. Phys.: Condens. Matter* 2019; **31**: 153001.

6. He W, Zhu T, Zhang X *et al*. Ultrafast demagnetization enhancement in CoFeB/MgO/CoFeB magnetic tunneling junction driven by spin tunneling current. *Sci. Rep.* 2013; **3**: 2883.

7. Longa D, [Kohlhepp](https://xueshu.baidu.com/s?wd=author%3A%28JT%20Kohlhepp%29%20&tn=SE_baiduxueshu_c1gjeupa&ie=utf-8&sc_f_para=sc_hilight%3Dperson) J-T, de [Jonge](https://xueshu.baidu.com/s?wd=author%3A%28WJMD%20Jonge%29%20&tn=SE_baiduxueshu_c1gjeupa&ie=utf-8&sc_f_para=sc_hilight%3Dperson) W-J-M *et al*. Influence of photon angular momentum on ultrafast demagnetization in nickel. *Phys. Rev. B* 2007; **75**: 224431.

8. Kaoru Y. *Progress in Ultrafast Intense Laser Science V*. Heidelberg: Springer Berlin, 2010.
